# Can we make Chitosan by Enzymatic Deacetylation of Chitin?

**DOI:** 10.3390/molecules24213862

**Published:** 2019-10-26

**Authors:** Rianne A. G. Harmsen, Tina R. Tuveng, Simen G. Antonsen, Vincent G. H. Eijsink, Morten Sørlie

**Affiliations:** Department of Chemistry, Biotechnology and Food Science, Norwegian University of Life Sciences, PO 5003, N-1432 Ås, Norwaytina.tuveng@nmbu.no (T.R.T.); simen.antonsen@nmbu.no (S.G.A.);

**Keywords:** chitin, chitosan, chitin deacetylase, nuclear magnetic resonance

## Abstract

Chitin, an insoluble linear polymer of β-1,4-*N*-acetyl-d-glucosamine (GlcNAc; A), can be converted to chitosan, a soluble heteropolymer of GlcNAc and d-glucosamine (GlcN; D) residues, by partial deacetylation. In nature, deacetylation of chitin is catalyzed by enzymes called chitin deacetylases (CDA) and it has been proposed that CDAs could be used to produce chitosan. In this work, we show that CDAs can remove up to approximately 10% of *N*-acetyl groups from two different (α and β) chitin nanofibers, but cannot produce chitosan.

## 1. Introduction

Chitin, the second most abundant biopolymer in nature, is a crystalline linear polysaccharide containing β-1,4 linked *N*-acetyl glucosamine (GlcNAc, A) residues. It is a common structural component in cell walls of yeast and fungi and in the exoskeletons of insects, crustaceans, and parasitic nematodes [1,2]. Alpha-chitin is the most common chitin form. It contains GlcNAc chains in an antiparallel orientation [3], and it is found in insects, crustaceans, fungi, and yeast. Beta-chitin is derived from squid pens and exists in parallel GlcNAc chains [1,4]. In γ-chitin, the rarest crystalline form of chitin found in insects and in the stomach of the Loligo squid, two GlcNAc chains are oriented in one direction and the third runs in the opposite direction [3,5]. The individual chitin chains are held together by hydrogen bonds [3].

Partial deacetylation of chitin yields chitosan, a heteropolymer of (1-4)-linked (GlcNAc) and glucosamine (GlcN, D) units with the GlcN units randomly distributed along the polymer chain [6]. The name chitosan refers to a continuum of soluble polymeric chitin derivatives that can be described and classified according to several characteristics where the degree of polymerization (DP) and the fraction of *N*-acetylated residues (F_A_) are the most important. In weak acidic media, the GlcN units are charged, making chitosan, unlike chitin, water-soluble [1,7]. Solubility in weak aqueous media requires the F_A_ to be below approximately 0.65 [8]. Due to its non-toxicity, biodegradability, and biocompatibility, combined with interesting biological and physicochemical properties, chitosan has various applications in agriculture, industry, medicine, water treatment, and biotechnology [1,7]. The most common method for conversion of chitin to chitosan is usually done by demineralization and deproteination of chitin-rich biomass at high temperature using aqueous NaOH as a base [9]. This is a relatively uncontrolled process that generates basic wastewater that may lead to environmental pollution [10].

In nature, enzymes called chitin deacetylases (CDAs) that belong to the family 4 of carbohydrate esterases (CE4), according to the CAZy classification system (www.cazy.org) [11], catalyze the removal of *N*-acetyl groups from chitin. This esterase family comprises enzymes that de-*N*- or de-*O*-acetylate chitin, acetyl xylan and peptidoglycan. Ever since the discovery of the characterization of these enzymes, it has been speculated that CDAs could be used to convert chitin into chitosan, in what would be an environmentally-friendly and potentially cost-efficient approach. While seemingly attractive, from a theoretical point of view, it may not seem very likely that CDAs would be able to solubilize chitin (further discussed below). In this study, we have investigated the possibility of several CDAs to solubilize chitin, focusing on a chitin deacetylase from *Vibrio cholerae* (*Vc*CDA) as a deacetylation catalyst [12]. As a substrate, we assessed both α- and β-chitin nanofibers with a high surface to volume ratio, which presumably would make these fibers particularly suited for enzymatic deacetylation.

## 2. Results and Discussion

Initially, three enzymes with known chitin-deacetylating activity, *Vc*CDA from *Vibrio cholera* [12,13], *An*CDA9 from *Aspergillus nidulans* [14], and *Sp*PgdA from *Streptococcus pneumoniae* [15] were assessed for their ability to deacetylate chitin nanofibers using the methods described below. Incubation with chitin nanofibers for 48 h displayed an ability of the deacetylases to remove 3–6 % of the *N*-acetyl groups (results not shown). For further investigation, we chose to focus on *Vc*CDA since this appeared to most efficient of the three enzymes.

**Figure 1:**
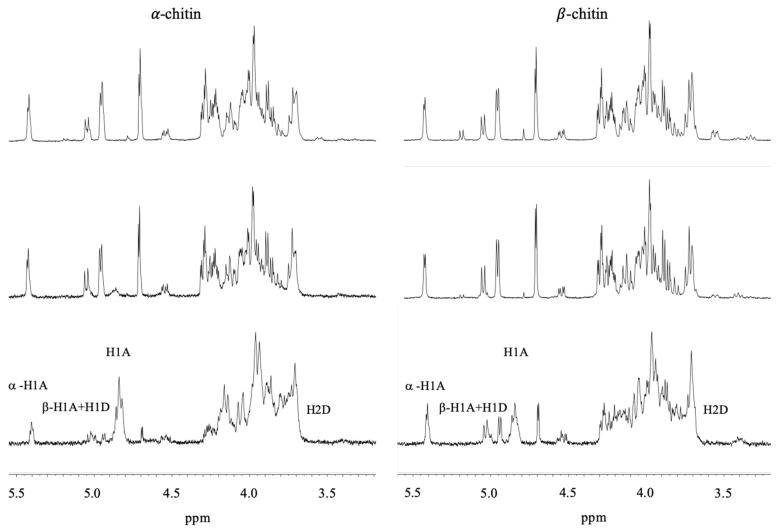
^1^H NMR spectrum of untreated α-chitin (bottom left, F_A_ = 0.950) and β-chitin (**bottom right**, F_A_ = 0.895) nanofibers before addition of *Vc*CDA and after 24 h (α-chitin **middle left**, F_A_ = 0.945) and (β-chitin **middle right**, F_A_ = 0.873), and 48 h (α-chitin **middle left**, F_A_ = 0.898) and (β-chitin **middle right**, F_A_ = 0.852) of incubation with *Vc*CDA. The peaks of βH1 and H1D come at ppm of 5.05 and 5.07, respectively, H1A comes at ppm 4.91, and H2D comes at ppm 3.44 [16].

**Figure 2:**
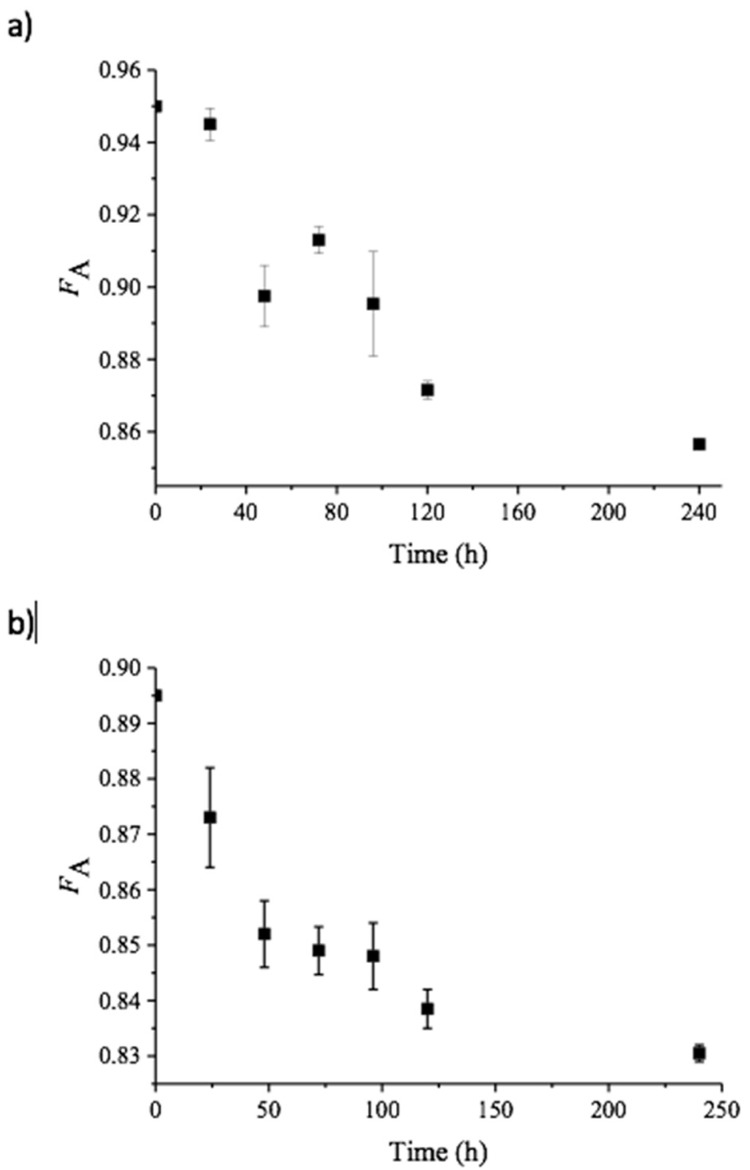
Change in F_A_ over time upon incubation of α-chitin (**a**) or β-chitin (**b**) with *Vc*CDA at pH 8.0 and t = 37 °C, as determined by ^1^H NMR spectroscopy. The points show average values derived from three independent experiments with standard deviations. Note that starting F_A_ values were 0.950 and 0.895 for α- and β-chitin, respectively.

Prior to in-depth studies with *Vc*CDA, untreated α- and β-chitin nanofibers were subjected to ^1^H NMR to determine the F_A_ of the starting materials according to Einbu and Vårum (Figure 1) [16]. The F_A_ was calculated using Equation (1):

(1) $$F_{A}\;=\;\frac{\left(I_{\alpha H1A}\;+\;I_{\beta H1A+H1D}\;+\;I_{H1A}\right)-I_{H2D}}{\left(I_{\alpha H1A}\;+\;I_{\beta H1A+H1D}\;+\;I_{H1A}\right)},$$

where I_αH1A_ represent the integral of the H-1 protons of the α-anomer at the reducing end of GlcNAc residues, I_βH1A+H1D_ represents the integral of the H-1 protons of the β-anomer at the reducing end of GlcNAc and GlcN residues, respectively, I_H1A_ represents the integral of H-1 protons of GlcNAc residues within the polymer chain, and I_H2D_ represents the integral of H-2 protons of GlcN residues with the polymer chain [16]. By subtracting the integral from H-2 from GlcN units (I_H2D_) from the integral representing all H-1 protons (I_αH1A_ + I_βH1A+H1D_ + I_H1A_), the integral representing only acetylated unit is obtained. Thus, Equation (1) implies that the F_A_ is determined by dividing the integral of only acetylated units by the integral of all H-1 protons [16]. As expected for polymeric chitin, no significant peak was observed for the α-conformation at the C1. This approach yielded F_A_ values of 0.950 and 0.895 for α- and β-chitin, respectively (Figure 1). This implies that the chitin nanofibers contain an inherent small fraction of deacetylated units, in accordance with previous observations [16]. The fraction of acetylated units (F_A_) in both α- and β-chitin nanofibers after incubation with *Vc*CDA at 37 °C was determined at different time points (Figure 2). After 10 days, the F_A_ values reached minima of 0.85, for α-chitin, and 0.83 for β-chitin as observed as a stopped decline in F_A_ values with respect to time (depicted in Figure 2).

To ensure that the decrease in the rate of deacetylation was not due to the inactivation of *Vc*CDA. A new batch of the enzyme was added after 240 h, thus doubling the total amount of added enzyme from 0.5 µM to 1.0 µM and incubated for another 24 h. This did not lead to a significant further increase in deacetylation (F_A_ of 0.857 vs. 0.854 for α-chitin and 0.831 vs. 0.824 for β-chitin, respectively), indicating that essentially no more acetyl groups are available as a substrate for *Vc*CDA.

**Figure 3:**
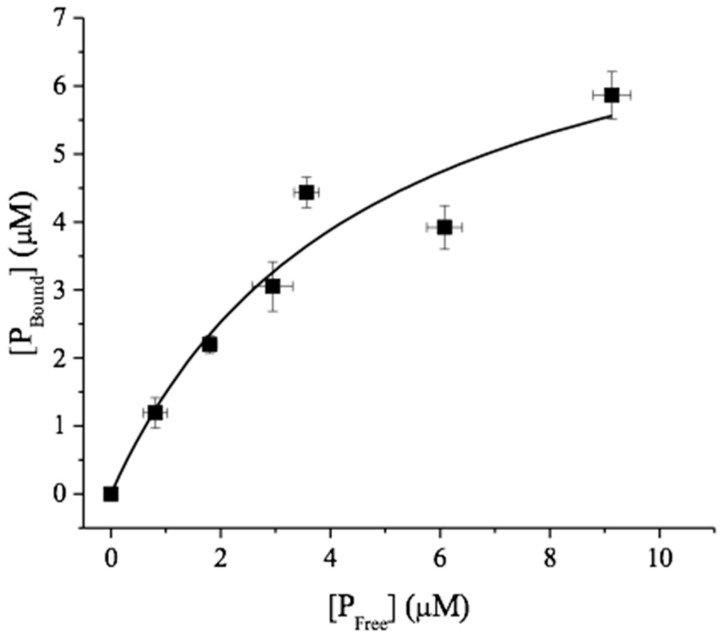
Equilibrium isotherm for the adsorption of *Vc*CDA to β-chitin nanofibers at pH 8.0 and t = 37 °C.

In another control experiment, soluble *N*,*N*-diacetyl chitobiose (to a final concentration of 1 mM) was added to the reaction mixtures after 120 h of incubation with the chitin substrates and was allowed to incubate for 24 h with the chitobiose substrate. *Vc*CDA catalyzes the hydrolysis of the *N*-acetyl groups attached to the penultimate GlcNAc residue from the non-reducing end [13], and mass spectrometry analysis showed that 24 h after its addition, all chitobiose had lost an *N*-acetyl group (Figure S1). It is thus clear that the stagnation in the deacetylation is not due to enzyme deactivation. The binding of *Vc*CDA to β-chitin nanofibers was also assessed to see if the low degree of maximum deacetylation could be due to strong, perhaps unproductive binding of *Vc*CDA to the substrate, which could make the enzyme incapable of performing catalysis. The plot of bound protein versus free protein was fitted to Equation (2) yielding a K_d_ of 5 ± 2 µM and a B_max_ of 8.4 μM, in the fit, which corresponds to 0.1 μmol/gram chitin fiber (Figure 3).

(2) $$P_{\mathrm{bound}}\;=\;\frac{B_{\max}\;\times \;\left[P_{\mathrm{free}}\right]}{K_{d}\;+\;\left[P_{\mathrm{free}}\right]},$$

This suggests that, at conditions used with an initial concentration of VcCDA of 0.5 μM, 0.18 μM will at any time be free in solution and 0.32 μM associated with the substrate. It is thus not likely that the non-productive binding of *Vc*CDA led to reduced deacetylation.

## 3. Materials and Methods

### 3.1. Materials

Both α- and β-chitin nanofibers, derived from crab shells, were kindly donated by S. Ifuku, Department of Chemistry and Biotechnology, Tottori University Japan (Tottori City, Japan) [17,18]. Dialyzed chitin nanofibers DH_2_O were lyophilized using a Christ Alpha 2-4 LD plus freeze dryer. *N*,*N*-Diacetyl chitobiose was purchased from Megazyme (Wicklow, Ireland). All further chemicals and solvents were purchased from Sigma-Aldrich (St. Louis, MO, USA) and used without further purification.

### 3.2. Production of AnCDA9, SpPgdA, and VcCDA

*An*CDA9 from *Aspergillus nidulans* was produced in *Escherichia coli* and purified as described previously [14]. *Sp*PgdA from *Streptococcus pneumoniae* in *E. coli* and purified as described previously [15].

The gene encoding *Vc*CDA (accession code AAF94439) was ordered from Gen-Script (Piscataway, NJ, USA). The gene, without the nucleotides encoding the signal peptide, was amplified by PCR using a forward (5′-TTAAGAAGGAGATATACTATGAATAGCACCCCGAAAGGCA C-3′) and reverse (5′-AATGGTGGTGATGATGGTGCGCCAGCGCGGTGAACAGGGTA-3′) primer (Eurofins, Ebersberg, Germany). The underlined nucleotides represent over-hang sequences. The amplified PCR product was subsequently cloned into the pNIC-CH vector [19] utilizing ligation-independent cloning (LIC) [20]. As a result of this cloning strategy, the N-terminus of the (signal peptide-free) protein was extended with methionine on the N-terminus, while a seven residue His-tag (AHHHHHH) was added at the C-terminus. The pNIC-CH vector containing the *Vc*CDA gene sequence was then transformed into chemically competent XL1-Blue cells (Agilent, CA, USA) by heat shock. The host strain was allowed to proliferate in Super Optimal broth with catabolite repression (SOC) for 60 min prior to plating on lysogenic broth (LB) agar containing 50 µg/mL kanamycin and 5% sucrose. After incubation overnight at 37 °C, single transformant colonies were inoculated in liquid LB containing 50 µg/mL kanamycin and incubated overnight at 37 °C. The plasmid was isolated from transformants using a NucleoSpin Plasmid kit (Macherey-Nagel), and the gene sequence of *Vc*CDA was verified by Sanger sequencing (GATC, Konstanz, Germany). The isolated plasmid was subsequently transformed by heat shock into chemically competent OneShot BL-21 Star^TM^ (DE3) *E. coli* cells (Invitrogen) and grown in SOC media as described above, before plating on LB agar containing 50 µg/mL kanamycin and overnight incubation at 37 °C. A transformant colony was inoculated in 5 mL LB (50 µg/mL kanamycin) and incubated at 37 °C overnight. The pre-culture was then grown in 0.5 L Terrific Brothcontaining 50 µg/mL kanamycin using a Harbinger system (Harbinger Biotechnology and Engineering, Markham, Canada) at 21 °C. After 24 h, the culture was induced with isopropyl-β-d-thiogalactopyranoside (final concentration 0.2 mM), followed by incubation at 21 °C for 24 h. The cells were harvested by centrifugation (8000 rpm, 20 min at 4 °C) and the pellet resuspended in 30 mL 20 mM Tris-HCl pH 8, 20 mM imidazole, 500 mM NaCl before the cells were lysed using a Vibra-cell sonicator (Sonics and Materials Inc., Newtown, CT, USA) with 5 s on/off pulses for 4 min at 28% amplitude while kept on ice. The cell debris was removed by centrifugation at 12,000 rpm for 15 min and the cell-free protein extract was filtrated using a 0.45 µm syringe filter (Sarstedt, Nümbrecht, Germany). Proteins were purified from the resulting cell-free extract using a column packed with Ni-NTA Agarose (Qiagen, Venlo, The Netherlands, 1.5 cm in diameter, 5 mL stationary phase in total). The column was pre-equilibrated in a buffer containing 20 mM Tris-HCl, 20 mM imidazole, and 500 mM NaCl, pH 8.0, before the cell extracts were applied. After washing with a buffer containing 20 mM Tris-HCl and 500 mM NaCl, pH 8.0, the enzyme was eluted with a buffer containing 20 mM Tris-HCl, 250 mM imidazole, and 500 mM NaCl, pH 8.0. A flow rate of 2.5 mL/min was used at all times. Enzyme purity of all CDAs was verified by SDS-PAGE (Supplementary Figure S1) and fractions containing purified enzymes were concentrated and transferred to 20 mM potassium phosphate buffer pH 6.0 by centrifugal ultrafiltration (Macrosep Advance Centrifugal Device, 10 kDa cutoff, Pall Corporation, Port Washington, NY, USA). Protein concentration was determined by using the Bradford Protein Assay from Bio-Rad (Hercules, CA, USA).

After purification, the enzymes were checked to have similar activity as reported values [13,14,15] by initial acetate release from *N*,*N*-diacetyl chitobiose (1 mM) measured by IC using a Dionex ICS3000 system with suppressed conductivity detection as described by Liu et al. [14].

### 3.3. Enzymatic Deacetylation of Chitin Nanofibers

Both α- and β-chitin nanofibers (5 mg/mL) were incubated with CDA (0.5 µM) in Tris-HCl (25 mM, pH 8.0) and CoCl_2_ (0.1 mM) at 37 °C, with shaking at 800 rpm. Samples (13 mL) were taken at different time points and enzyme activity was immediately quenched by adding an equal volume of acetonitrile. After removal of the organic solvent under reduced pressure, the materials were dialyzed to H_2_O, using Spectra/Por 6 dialysis membranes with a cutoff of 100 Da, followed by lyophilization using a Christ Alpha 2–4 LD plus freeze dryer, which rendered the chitin fibers colorless solids. Three parallels of each sample were prepared for ^1^H NMR analysis.

### 3.4. ^1^H NMR of α- and β-Chitin Nanofibers

^1^H NMR sample preparation was conducted as described by Einbu and Vårum [16]. The lyophilized chitin nanofiber samples were first wetted with 100 µL 1% DCl in D_2_O (99.9 atom % D, contains 0.05 wt% TMS as internal standard) followed by dissolving in 700 µL 37% DCl. ^1^H NMR spectra were recorded at 25 °C with 64 scans using a Bruker AscendTM 400 MHz spectrometer. The chemical shifts are reported in parts per million (ppm) relative to TMS.

The fraction of acetylated units (F_A_) was calculated according to Einbu and Vårum [16] using the integrals of H1- and H2 protons of GlcNAc and GlcN residues as described in the text.

### 3.5. Enzyme Activity Test

Remaining chitin deacetylase activity after five days of incubation with chitin nanofibers was measured by adding of *N*,*N*-diacetyl chitobiose to the reaction mixture to a final concentration of 1 mM. After 24 h, deacetylation products were analyzed by MALDI-TOF, as described by Cederkvist et al. [21].

### 3.6. Binding Assay

Enzyme binding to β-chitin nanofibers was assessed as described by Zakariassen et al. [22]. *Vc*CDA was diluted to various concentrations (0–20 µM) in Tris-HCl (25 mM, pH 8.0) and 0.1 mM CoCl_2_. The A_280_ of these solutions was measured, and a standard curve was created. The reaction volume was 1.0 mL, the concentration of β-chitin WAS 5 mg/mL, and the *Vc*CDA concentration in the mixtures was 0, 2, 4, 6, 8, 10, and16 µM, respectively. The mixtures were incubated at 37 °C and 300 rpm. After 2 h, the β-chitin nanofibers were spun down for 20 min at 13,000 rpm, and the A_280_ values of the supernatants were measured. The bound and free protein concentrations ([P_bound_], [P_free_]) were calculated from the standard curve. All assays were performed in triplicate. The equilibrium dissociation constant K_d_ (µM) and binding capacity B_max_ (µmol/g) were determined by fitting the binding isotherms to the following equation: [P_bound_] = B_max_ × [P_free_]/[K_d_ + P_free_] by non-linear regression using Origin v7.0 software (OriginLab Corporation, Northampton, MA, USA).

## 4. Conclusions

Polymers of chitin are synthesized as crystalline fibrils that are hundreds to thousands of monomer units long [23]. Calculations show that the removal of a GlcNAc dimer from chitin crystal comes with a thermodynamic penalty of 8 kcal/mol [24]. Our results demonstrate that a CDA is able to reduce the degree of acetylation for two different types of crystalline chitin (α and β), but only by up to approximately 10%. This prompts us to speculate that the enzymatic action of a CDA is not able to overcome the thermodynamic penalty of removing single polymer chains from the crystal to access all available *N*-acetyl groups. In nature, chitin is degraded by glycoside hydrolases (GH) and lytic polysaccharide monooxygenases (LPMO) [25,26]. GHs overcome the thermodynamic penalty of decrystallization by strong binding to a single polysaccharide chain through several surface-exposed aromatic amino acids and by this pulling the chain from the crystal into the active site of the enzymes [27,28]. Moreover, the binding affinity of GHs towards the substrate increases with an increasing chain length of the substrate [29,30]. Typical binding free energy of a chitin active GH on six GlcNAc residues is −9 kcal/mol (*K*_d_ = 0.2 μM) [28]. LPMOs achieve decrystallization by activating an oxygen species on its copper-active site that is calculated to be strong enough, oxidize a C-H bond of 110 kcal/mol [31]. In contrast, we observe that *Vc*CDA binding free energy to β-chitin of −7.5 kcal/mol (*K*_d_ = 5 μM). Such a relative weak binding affinity is also confirmed from obtained Michaelis–Menten constants from various kinetic analyses. *Sp*PgdA is reported to have a *K*_m_ of 3.8 mM, which translates to a binding free energy of −3.4 kcal/mol, with (GlcNAc)_3_ as the substrate, while *An*CDA9 has a *K*_m_ of 72 μM, which translates to a binding free energy of −5.9 kcal/mol, with (GlcNAc)_5_ as the substrate [14,15].

Even though it is not likely that the use of a CDA alone is sufficient to produce chitosan directly from chitin, its action on crystalline chitin may be useful. CDA action introduced NH_2_-group on the fibril surface, which can serve as a starting point for surface modifications, i.e., through “click chemistry”, thus offering an opportunity for creating new materials [32,33].

## Supplementary Materials

The following are available online, Figure S1: MALDI-TOF-MS spectra of *N,N*-diacetyl chitobiose after incubation with *Vc*CDA for 24 h. Figure S2: Table S1: SDS PAGE GEL analysis of *Vc*CDA, *Sp*PgdA, and *An*CDA9.
