# Supplementary material for: Can we make Chitosan by Enzymatic Deacetylation of Chitin?
Source: Molecules. 2019 Oct 26;24(21):3862. doi: 10.3390/molecules24213862 (PMC6864559; doi:10.3390/molecules24213862)
Supplement: Supplementary file 1 [file molecules-24-03862-s001.pdf]

Can we make chitosan by enzymatic deacetylation of chitin?

Rianne A.G. Harmsen, Tina R. Tuveng, Vincent G.H. Eijsink, and Morten Sørlie*

Department of Chemistry, Biotechnology and Food Science, Norwegian University of Life Sciences, PO 5003, N-1432 Ås, Norway.

* To whom correspondence should be addressed, Morten Sørlie (morten.sorlie@nmbu.no)

Keywords: Chitin; chitosan; chitin deacetylase; nuclear magnetic resonance.

Table of Contents:

Figure S1. MALDI-TOF-MS spectra of *N*,*N*-diacetyl chitobiose after incubation with *Vc*CDA for 24 hours.

Figure S2. SDS PAGE GEL analysis of *Vc*CDA, *Sp*PgdA, and *An*CDA9. 3

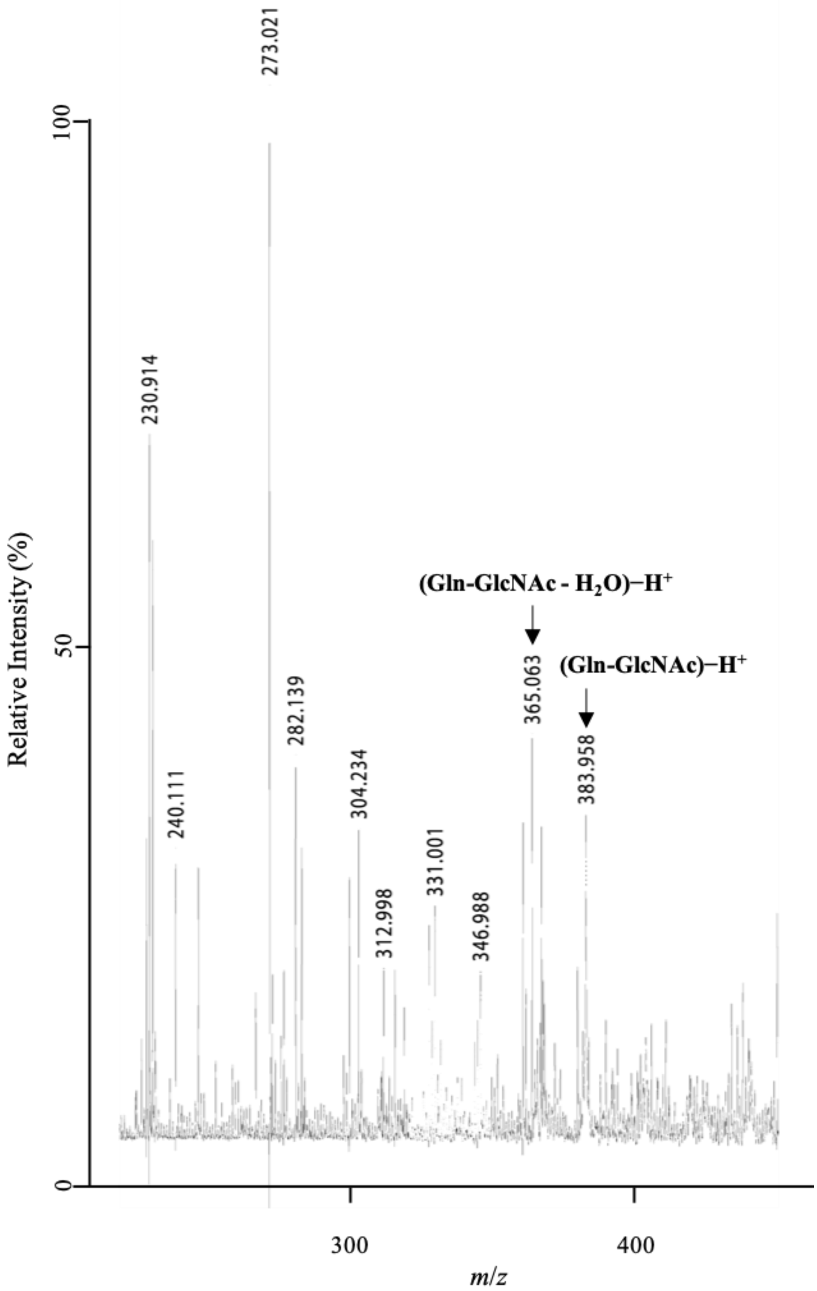

Figure S1. MALDI-TOF-MS spectra of *N*,*N*-diacetyl chitobiose after incubation with *Vc*CDA for 24 hours. We observe protonated GlcN-GlcNAc at *m/z* of 383 and protonated GlcN-GlcNAc minus H_2O at *m/z* of 365 indicated by arrows. There are no significant peak at *m/z* of 425 corresponding to protonated GlcNAc-GlcNAc.

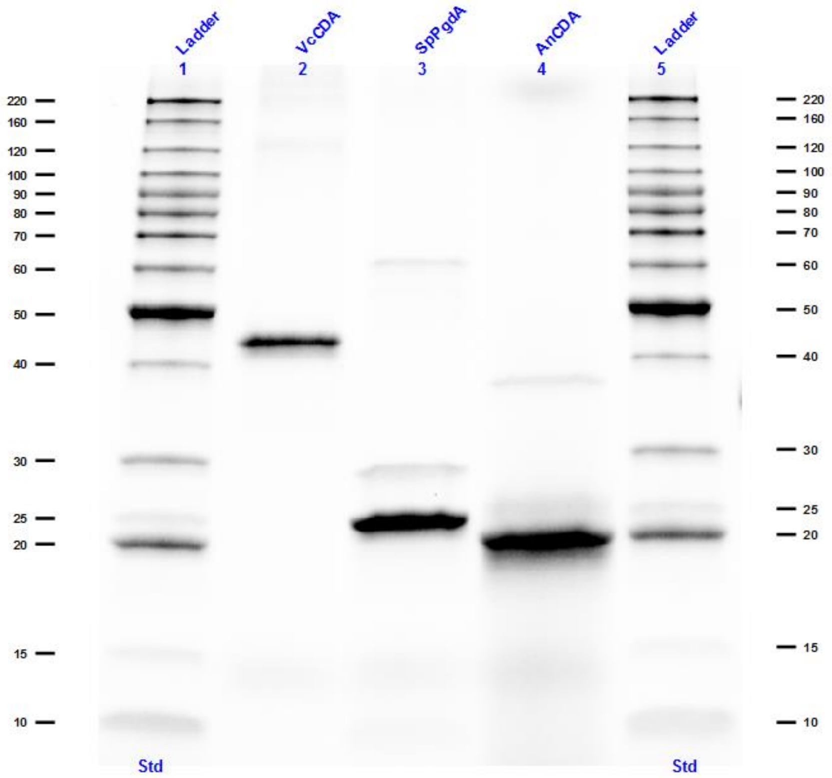

Figure S2. SDS–PAGE GEL analysis of *Vc*CDA, *Sp*PgdA, and *An*CDA9. Lane 1, protein standard; lane 2, *Vc*CDA; lane 3, *Sp*PgdA, and lane 4, *An*CDA9.
